# Development and validation of the Chinese mental health value scale: a tool for culturally-informed psychological assessment

**DOI:** 10.3389/fpsyg.2025.1417443

**Published:** 2025-03-24

**Authors:** Yujia Lei, Changming Duan, Kehan Shen

**Affiliations:** ^1^Center for Counseling & Psychological Services, Washington University in St. Louis, St. Louis, MO, United States; ^2^Department of Educational Psychology, University of Kansas, Lawrence, KS, United States; ^3^Counseling and Psychological Services, University of California, Berkeley, Berkeley, CA, United States

**Keywords:** mental health, values, Chinese culture, college students, scale development, wellbeing

## Abstract

**Background:**

Mental health values play a significant role in defining, promoting, and intervening in mental health. As part of an individual’s value system, these values are inherently cultural. However, they remain underexplored in Chinese culture context, particularly among the large young adult population of university students. A culturally informed tool to assess mental health values is much needed to support China’s efforts in promoting mental health among college students.

**Methods:**

Four steps were taken to complete the Chinese Mental Health Value Scale (CMHVS) development, namely, item pool construction including expert reviews, a pilot study for item revision and selection, data collection for explorative and confirmatory factor analyses, and validity testing.

**Results:**

A 35-item, seven-factor model was identified with high reliability (Cronbach’s alpha of 0.96), and evidence for convergent validity. The seven factors were *Expected Self, Relating to Others, Life Principles, Family, Purpose and Meaning, Achievement*, and *Communication*.

**Conclusion:**

The CMHVS provides a culturally grounded method for assessing mental health values in Chinese university students. It has potential applications in research and clinical settings, improving culturally sensitive mental health promotion and intervention.

## Introduction

The recent paradigm shift from the traditional healthcare model to population health perspective promoted by American Psychological Association (APA) as an organizing framework (Evans, 2021) repositions psychology’s roles in health care and health equity enhancement. This shift also revalidates the call for focusing on mental health (vs. mental illness only) in health care (Haeyen et al., 2018). This was consistent with the two continua model of mental health and illness (Keyes, 2014), which highlighted the distinctive features of mental illness and mental health. The model called for more investigation of mental health (as opposed to mental illness), and provided a refreshing perspective on enhancing preventive and interventive efforts to address the global mental health crisis (Patel et al., 2018). The understanding that promoting mental health may decrease the negative impact of mental illness is consistent with the Eastern *Taiji* philosophy of *Yin* and *Yang* (Scheid, 2016), which underlies Chinese traditional medicine and views mental health and illness as two opposite as well as complementary energies. Strengthening mental health is an essential aspect of reducing mental illness. Understanding mental health values is a critical step toward fostering value-consistent healthy behaviors, mindsets, and overall wellbeing.

Unfortunately, mental health and mental illness are often used as exchangeable terms in both the day-to-day language and professional communications in the western societies (Benton, 2018), which unavoidably leads to decreased attention to mental health in face of mental illness challenges. The sobering fact is, however, that the de facto clinical treatment of mental illness by and large has not yielded the desired outcome of eliminating or even reducing mental illness (Keyes, 2014). Thus, it is time that we reexamine what mental health is and how it can be promoted as a part of the effort to remedy the crisis due to increased mental illness. It becomes concerning if we consider the degree to which mental illness theories and intervention methods based on western medical models have been transported to many parts of the world (Duan, 2019). For instance, western models have strongly influenced mental health education and clinical practice in China (Jia, 2016). Diagnostic labels for mental illnesses have become widely publicized and have entered public awareness, particularly among young adults. This trend led to the intensified debate in recent years regarding the cultural fit of western models and the consequence of overlooking core Chinese values like family, relational harmony, and implicit communication. Thus, it is imperative that the awareness be raised that the effectiveness of mental illness interventions for specific populations rely on the success in mental health promotion, which must be grounded in an understanding the mental health values specific to the cultural contexts.

In this study, we examined and evaluated the mental health values of Chinese college students, a population estimated to comprise of 30.32 million undergraduate and 7.6 million graduate students in 2019 in China (Textor, 2021) and 703,500 overseas (Ministry of Education, 2020). Represent a crucial demographic in China, their mental health is a growing public concern, particularly given academic stress, family and cultural expectations regarding achievement. Presently, mental health interventions for this population both in China and overseas are largely prescribed by western theories and systems (Duan, 2019). The issue of cultural fit is a serious one as mental illnesses “are every bit as shaped and influenced by cultural beliefs and expectations” (Watters, 2011, p. 4). Scholars and practitioners should be reminded that effective counseling is to facilitate psychological growth and bring positive therapeutic changes defined in a given cultural context (Wampold, 2001). Understanding Chinese college students’ mental health values in a Chinese cultural context can thus inform more effective, culturally sensitive interventions. Moreover, most current mental health scales on Chinese students are based on psychopathology, while this study aimed to assess from mental health, a different perspective that offered a full picture based on the two continua model (Keyes, 2014).

### Mental health, values, and culture

Defining mental health has not been easy, partly due to the tangle between mental health and mental illness conceptions, and partly the cultural nature of the construct. The World Health Organization (WHO) provides a broad definition containing “a state of well-being in which an individual realizes his or her own abilities, can cope with the normal stresses of life, can work productively and is able to make a contribution to his or her community” (World Health Organization, 2018, p. 1). This definition challenges western medical model based clinical practice and draws attention to both mental illness reduction and mental health enhancement. Moreover, the WHO’s conceptualization makes it clear that mental health is a culture-determined phenomenon (Bains, 2005).

Cultural values determine personality and pathology (Marsella and Yamada, 2000) and influence how mental health is viewed, assessed, and promoted (Jensen and Bergin, 1988; Tyler et al., 1983). Understanding cultural specific mental health values “as orienting beliefs about what is good or bad for clients and how that good can be achieved” (Jensen and Bergin, 1988; p. 290) is critical in combatting mental illness and promoting health. In a global context, mental health services cannot ignore the differences in values across countries and cultures as they reflect individuals’ interaction with their cultural contexts (World Health Organization, 2004).

### Chinese cultural context

Traditional cultural values rooted in Confucianism, Taoism, and Buddhism are built into people’s deep structure of mind and shape psychology (Hwang, 2012). Distinctive cultural features include a supreme emphasis on family (*jia*, 家), relationship (*guanxi*, 关系), and harmony (*he*, 和), which leads the culture being viewed as collectivistic and relational in nature (Gold et al., 2002). The Chinese *wo* (我—I or Me in English), the source of the idea of the “self,” for instance, is defined in relational terms and tied to the family (Lin, 2001). Thus, Filial Piety (*xiao*, 孝), an ancient Chinese philosophical concept, has remained an important moral tenet that expects children to offer love, respect, support, and deference to their parents and other elders in the family. Moreover, due to the paramount importance placed in relationship, family, and harmony, the society values the individual self as being field-dependent, contextual, relationship/family/others-oriented, holistic, interdependent, social-centric, and authoritarian-oriented, which sharply contrasts the individualistic definition of healthy self as field-independent, separated, unique, self-sufficient, egocentric/auto-centric, self-absorbed, and egalitarian (Yang, 2003). Li et al. (2019) found empirical evidence that Chinese tended to have an interdependent self-construal that was positively associated with relational self-esteem (vs. personal self-esteem). Liu and Goto (2007) revealed a significant relationship between self-construal and mental distress and family relations among Asian American adolescents.

Chinese culture is viewed being high in context, placing high importance of context in interpersonal relationship and communication (Hall, 1976). People tent to see themselves as part of networks of friends and family, and are socialized to be observant of others’ reactions to the self. In communication, speakers rely on context information other than mere words to convey meaning they intend to deliver (Kim et al., 1998), and show interpersonal sensitivity and use feelings to guide behavior (Gudykunst, 2001). As the result, implicit communication (*hanxu*, 含蓄), listening-centered communication (*ting hua*, 听话), polite communication (*ke qi*, 客气), and insider/in group-communication (*zi ji ren*, 自己人) are highly valued. As Gao (1998) stated, “the primary functions of [Chinese] communication are to maintain existing relationships among individuals, reinforce role and status differences, and to preserve harmony within the group” (p. 168).

### Empirical research on mental health values

Only a few early studies are available in the English literature on mental health values, not including those focusing on specific religious/spiritual values. The Mental Health Values Questionnaire (MHVQ) developed by Tyler et al. (1983) measured a person’s conception of good mental health in self-acceptance, negative traits, achievement, affective control, good interpersonal relationships, untrustworthiness, religious commitment, and receptivity to unconventional experiences. Tyler et al. (1989) showed that the degree of agreement between counselor and patient on several MHVQ subscales had a positive influence in therapeutic outcomes in treatment of inpatient chemical dependency clients. To examine its cross-cultural utility, Tyler and Suan (1990) discovered that Japanese American undergraduate students scored higher on good interpersonal relations, trustworthiness, and absence of negative personal traits than Caucasian American students.

Jensen and Bergin (1988) surveyed a large group of psychologists, therapists, social workers, and psychiatrists (94% Caucasian, 60% male), using a questionnaire developed for the study. They found a consensus that certain basic values are important for defining mentally healthy lifestyles and for guiding and evaluating psychotherapy. These values include, in order of importance assigned: competent perception and expression of feelings, freedom/autonomy/responsibility, integration/coping ability, self-awareness/growth, human relatedness/interpersonal and family commitment, self-maintenance and physical fitness, mature values, forgiveness, regulated sexual fulfillment, and spirituality/religiosity. A few years later, Kelly (1995) developed their Mental Health Value Survey for members of American Counseling Association. They found seven highly endorsed values, namely, positive human relatedness, compassionate responsiveness, responsible self-expression, forgiveness, autonomy, purposeful personal development, and sexual acceptance. They also found differences associated with age, gender, and theoretical orientations on some of the values as well.

### The present study

The present study sought to highlight the importance and cultural nature of mental health values by: (1) developing a Mental Health Value Scale for use with Chinese college students; and (2) validating the scale by investigating the relationships between mental health values and broad cultural values (individualism–collectivism and Asian cultural values) and psychological wellbeing (life satisfaction and psychological stress).

## Methods

### Scale development

Following the scale development procedures outlined by DeVellis (2011), we took the following four steps to complete the Chinese Mental Health Value Scale (CMHVS) development. Informed consent was appropriately obtained from participants in each step. We used expert review (Chinese psychologists working and training in the U.S. and visiting scholars from China) at each step to minimize the interference of researcher biases.

**Step 1**. To build an item pool, we first did a thorough review of the literature on Chinese cultural values. Then we conducted a brief survey through email to students (*N* = 96) from a university in Beijing. The students were asked to describe characteristics of people they perceive as mentally healthy or unhealthy. The first author and another Chinese doctoral candidate in Educational Psychology reviewed and coded the responses and obtained seven domains with 16 sub-domains to reflect Chinese mental health values. Next, we conducted two focus groups, one with three Chinese professionals who had at least 1 year of clinical experience working with college students, and one with five college students. Participants shared their views and perceptions regarding “what matters and what is important?” in mental health. The first author transcribed and coded the transcripts and obtained 154 items in eight domains with 19 sub-domains. Lastly, a panel of four Chinese expert reviewers reviewed the items independently before a group decision was made to use the selected 102 items describing eight domains in the pool. The eight domains were Balance, Relationship, Emotion, Capacity, Following Norms, Self, Philosophy of Life, and Modernization.

**Step 2**. To evaluate and improve prospective items, we conducted a pilot study by asking participants to rate the 102 items through a Qualtrics survey. Sixty-seven Chinese college students took the survey and rated each item in both the importance to their mental health and to that of others. They were also asked to provide comments on the items if they had any. The result showed that all but eight items were perceived as important or very important to themselves and others. Then six Chinese doctoral students in counseling/clinical psychology did another round of review and edited the language based on participants’ comments on items. This process resulted in an improved version of CMHVS with 94 items (including 33 reversed items).

**Step 3**. We administered the 94 items along with a demographic questionnaire (and four scales for validity testing. See details in Step 4) to the targeted population. To establish the factor structure of CMHVS, an exploratory factor analysis (EFA) was conducted and followed by a confirmatory factor analysis (CFA). The data collection was done by sending the link of a Qualtrics survey either through social media *WeChat* or through collaborating colleagues in China for distribution.

Data were collected from 1,597 participants (Table 1). We deleted 223 cases that had more than 75% of the total responses missing and 289 cases for failure on the validity-check item (“Please choose ‘not important to me at all.’). The remaining missing values were replaced by the value of −999. To check for univariate outliers, z scores were examined (i.e., above 3.29) for each of the scale totals (Tabachnick and Fidell, 2012). Twelve cases above 3.29 were identified and treated as missing values and replaced with the Exception-Maximization technique. In addition, Mahalanobis distances among the variables were used to examine multivariate outliers, which resulted in five cases being deleted (probability of Mahalanobis D^2^ is less than 0.001). The remaining 1,058 cases were then randomly split into two data sets, Sample A (*n* = 529; Mage = 21.6, SDage = 3.0) and Sample B (*n* = 529; Mage = 21.6, SDage = 3.1). See Table 1 for detailed demographics of the samples. Then EFA was performed using Sample A and CFA Sample B. We randomly divided the sample to ensure that EFA and CFA used different samples.

**Table 1:** Participant demographics.

Characteristics	EFA		CFA	
	*n*	%	*n*	%
Gender				
Female	343	64.84%	365	69.00%
Male	186	35.16%	164	31.00%
Age				
18–22	351	66.35%	352	66.54%
23–26	152	28.73%	152	28.73%
27–30	16	3.02%	14	2.65%
30–40	10	1.89%	11	2.08%
Degree				
Associate degree	52	9.8%	57	10.78%
Undergraduate	298	56.33%	294	55.58%
Master’s	159	30.06%	165	31.19%
Doctoral	20	3.78%	14	2.65%
Grade				
Freshman	107	20.23%	112	21.17%
Sophomore	98	18.53%	90	17.13%
Junior	75	14.18%	69	13.04%
Senior	70	13.23%	80	15.12%
Department/Program				
Literal Arts	27	5.10%	30	5.67%
Science	69	13.04%	59	11.15%
Engineering	106	20.04%	109	20.60%
Philosophy	4	0.76%	7	01.32%
Medical	46	8.70%	51	9.64%
Law	42	7.94%	38	7.18%
Agriculture	2	0.38%	1	0.19%
Management	40	7.56%	67	12.67%
Economics	40	7.56%	35	6.62%
Education	66	12.48%	72	13.61%
Art	13	2.46%	14	2.65%
Others	45	8.51%	44	8.32%
Ethnicity/Race				
Han	491	92.82%	484	91.49%
Zhuang	10	1.89%	13	2.46%
Hui	5	0.95%	4	0.76
Man	4	0.76%	7	1.32%
Miao	3	0.57%	1	0.19%
Others	16	3.02%	20	3.78%
Religious beliefs				
No	476	89.98	490	92.63%
Yes	49	9.26%	37	6.99%
Missing	4	0.76%	3	0.57%
Only child				
Yes	226	42.72%	223	42.16%
No	303	57.28%	306	57.85%
Relationship status				
Single	319	60.30%	332	62.76%
Dating	194	36.68%	181	34.21%
Married	16	03.02%	16	03.02%

EFA, Exploratory factor analysis; CFA, confirmatory factor analysis.

**Step 4**. To obtain evidence of convergent validity, we conducted correlational analyses of CMHVS with four possible correlates, namely, individualism–collectivism orientation, Asian culture values, life satisfaction, and psychological stress. The following instruments were included in the data collection.

**Individualism and Collectivism Scale** (ICS; Triandis and Gelfand, 1998). This 16-item instrument was to measure individualism and collectivism values. It contains four subscales representing Horizontal Individualism (HI), Vertical Individualism (VI), Horizontal Collectivism (HC), and Vertical Collectivism (VC). Participants’ responses were recorded on a 9-point Likert scale, ranging from 1 (never or definitely no) to 9 (always or definitely yes). The scale showed good reliability in a Chinese student sample and the test–retest reliability is 0.75 (Huang et al., 2006).

**Asian Values Scale** (AVS; Kim et al., 1999) was designed to measure client adherence to Asian cultural values. AVS contains 24 statements reflecting Asian cultural values such as collectivism, conformity to norms, emotional self-control, family recognition through achievement, filial piety, and humility. Based on a Confirmative Factor Analysis we conducted in this study, only two subscales (Conformity to Norms and Humility) appeared to have adequate reliability (0.688 and 0.731). Participant responses were recorded by a 7-point Likert scale, ranging from 1 (strongly disagree) to 7 (strongly agree). The scale showed good test–retest reliability at 0.83 (Kim et al., 1999).

**Satisfaction with Life Scale** (SWLS; Diener et al., 1985) measures people’s general degree of satisfaction with their lives. It contains five items and uses a 7-point Likert scale, ranging from 1 (strongly agree) to 7 (strongly disagree). The internal consistency reported by Pavot and Diener (1993) was 0.85.

**The Brief Symptom Inventory-18** (BSI-18; Derogatis, 2000) was designed to measure psychological distress including depression, anxiety, and somatic symptoms. It consists of 18 symptoms experienced within the past 7 days and uses a 5-point Likert scale to record responses, ranging from 1 (not at all) to 5 (extremely). Scores can be obtained for anxiety, depression, and somatization individually in addition to the Global Severity Index (GSI). The BSI-18 has shown good internal consistency (0.92) for the GSI in Chinese samples (Wang and Mallinckrodt, 2006).

## Results

### Exploratory factor analysis

Exploratory factor analysis (EFA) with Sample A (*n* = 524) was conducted by the maximum likelihood estimation method in Mplus program (6th Version). A parallel analysis with all 94 items showed that two factors should be retained, with one being occupied by 32 out of 33 reversed items. Research suggested that reversed items often can create an artificial factor due to its wording (Kam, 2023). Given that the reverse items were randomly selected from the original item pool without any theoretical reasons, the 2-factor model was more likely to reflect a response pattern instead of item content. Additionally, a careful review showed that the contents of the reversed items were well represented by other non-reversed items. Therefore, we dropped the 33 reverse items and used the rest 61 items in the following analysis.

Table 2 presents indices of seven models, representing 1-, 2-, 3-, 4-, 5-, 6-, 7-factor models as Mplus stopped estimating after seven factors were extracted. The underlying dimensions of each model were carefully examined and compared to each other under the scrutiny of the expert panel (the same panel for the pilot study). The panel agreed that the 7-factor model could be adequately explained by the theoretical framework recognized in the literature review. The fit statistics supported that the 7-factor model as more parsimonious than other models as well.

**Table 2:** Fit statistics for EFA models with initial 61 items.

Model	*χ* ^2^	df	CFI	TLI	RMSEA	SRMR	AIC	BIC
Unstructured	5,819.05**	1,769	0.77	0.76	0.07	0.05	92,897.38	93,678.97
2 Factors	5,120.83**	1,709	0.81	0.79	0.06	0.05	92,319.16	93,357.01
3 Factors	4,445.93**	1,650	0.84	0.82	0.06	0.04	91,762.25	93,052.09
4 Factors	3,830.55**	1,592	0.87	0.85	0.05	0.03	91,262.87	92,800.43
5 Factors	3,523.46**	1,535	0.89	0.87	0.05	0.03	91,069.79	92,850.79
6 Factors	3,201.58**	1,479	0.90	0.88	0.05	0.03	90,859.91	92,880.09
7 Factors	2,902.64**	1,424	0.92	0.89	0.04	0.03	90,670.97	92,926.05

**p < 0.001. CFI, Comparative Fit Index; TLI, Tucker-Lewis Index; RMSEA, Root Mean Square Error of Approximation; SRMR, Standardized Root Mean Square Residual; AIC, Akaike; BIC, Bayesian.

In making item retention decisions, we used three criteria: (a) loading at least 0.40 on one factor, only allowing items with theoretical significance when the loading is slightly lower, (b) cross-loadings not exceeding 0.30, and (c) retaining factors that each had at least three items (Kahn, 2006). Based on the 7-factor model, seven items with low loadings (less than 0.40 on any factor) were deleted. A repeated EFA with the remaining items (Kahn, 2006) resulted in three additional items with loadings less than 0.40 and two items with cross-loadings higher than 0.30 deleted. Subsequently, the scale was modified through the same iterative process of deleting the five weakest items and three cross loaded items. In addition, the meaning of each item was examined, and the six items that were deemed unfit to the belonging factor were removed. It was worth noting that all the 26 items were removed one at a time and the EFAs were run multiple times until the final model is reached.

Good model fit indices were achieved for the final scale that contains 35 items loaded on seven factors, *χ*^2^ (371) = 709.12, CFI = 0.96, TLI = 0.94, SRMR = 0.02, RMSEA = 0.04, 90% confidence interval (0.04, 0.05). Our expert panel also deemed the seven factors as conceptually distinctive and theoretically sound. Although loadings of four items (#17, #18, #29, #35) were slightly below 0.40, they were retained in the scale for two reasons: (1) the theoretical framework strongly supported their existence; (2) their loadings were distinctively higher on the factor than other factors. Table 3 presents the seven factors and their respective items with factor loadings.

**Table 3:** Items, factor loadings, means and standard deviations.

Factors and items	1	2	3	4	5	6	7	M	SD
Factor 1: Expected self									
1. Being aware of one’s own negative emotions	**0.64**	−0.01	−0.02	−0.02	−0.01	0.02	−0.01	5.2	1.2
2. Adjusting to changes at different life and developmental stages	**0.61**	−0.00	−0.05	−0.01	0.02	0.08	−0.01	5.5	1.2
3. Taking responsibility for the consequences of one’s choices	**0.54**	0.13	0.04	0.04	−0.03	−0.06	0.07	5.6	1.2
4. Balancing physical and mental wellbeing	**0.53**	−0.01	0.12	−0.06	0.09	−0.05	0.03	5.6	1.2
5. Being able to see things from others’ perspectives	**0.51**	0.06	0.06	0.09	−0.06	0.03	0.04	5.6	1.2
6. Getting along well with others	**0.49**	−0.01	−0.01	0.16	0.02	0.20	0.03	5.7	1.1
7. Accepting life’s adversities	**0.47**	0.10	0.09	−0.09	0.01	−0.07	0.02	5.6	1.2
8. Maintaining normal functioning in daily life activities such as sleeping, eating, working, and studying	**0.45**	−0.04	−0.02	0.19	0.04	0.12	0.01	5.5	1.3
9. Being able to control one’s negative emotions	**0.44**	0.16	0.04	−0.05	0.17	−0.08	0.12	5.4	1.2
10. Maintaining harmony in interpersonal relationships	**0.42**	0.09	0.04	0.24	−0.03	0.06	0.03	5.5	1.2
11. Balancing one’s private life and social life	**0.40**	0.00	0.03	0.10	0.11	0.28	−0.03	5.0	1.3
Factor 2: Relating to others									
12. Bringing positive energy to others	−0.04	**0.76**	0.02	−0.08	0.01	0.2	−0.00	5.5	1.2
13. Helping people in need	0.01	**0.63**	0.13	0.04	−0.04	0.07	0.05	5.3	1.1
14. Having a peaceful mind	0.15	**0.55**	−0.01	0.08	0.16	0.01	−0.00	5.7	1.1
15. Being capable of loving others	0.18	**0.47**	−0.06	0.14	0.08	−0.02	−0.01	5.6	1.2
16. Feeling gratitude	−0.01	**0.45**	0.16	0.28	0.02	−0.09	0.09	5.8	1.1
17. Getting along well with people from different backgrounds (i.e., geographical, religious, ethnic, etc.)	0.28	**0.38**	0.03	0.06	0.05	0.07	−0.04	5.3	1.2
18. Having dialectical views of people and events	0.15	**0.35**	0.02	0.05	0.01	0.18	0.11	5.2	1.3
Factor 3: Life principles									
19. Being modest	−0.02	0.06	**0.90**	−0.00	0.02	−0.00	−0.02	5.2	1.3
20. Being prudent	0.06	0.04	**0.66**	0.02	0.02	0.12	0.06	5.2	1.3
21. Being neither arrogant about winning nor discouraged after losing	0.10	−0.07	**0.43**	0.06	0.28	0.08	0.04	5.2	1.3
Factor 4: Family									
22. Having healthy parents and family members	0.00	−0.03	0.14	**0.73**	0.05	−0.03	0.05	6.3	1.1
23. Having a harmonious family	−0.02	0.17	0.00	**0.60**	0.23	−0.04	−0.06	6.2	1.1
24. Showing filial piety to one’s parents	0.01	0.02	0.20	**0.56**	−0.05	0.19	−0.03	6.2	1.1
25. Having a sense of belonging in one’s family	0.07	0.20	−0.07	**0.50**	0.02	0.11	0.10	5.8	1.2
Factor 5: Purpose and meaning									
26. Having clear goals in life	−0.06	−0.01	−0.00	0.05	**0.80**	0.18	0.03	5.6	1.3
27. Being self-aware	0.08	0.09	0.03	−0.01	**0.75**	0.00	0.03	5.7	1.2
28. Feeling satisfied	0.12	0.24	0.07	0.01	**0.43**	−0.05	−0.04	5.3	1.3
29. Looking for meaning in life	0.07	0.23	−0.02	−0.02	**0.36**	0.15	0.13	5.5	1.2
Factor 6: Achievement									
30. Seeking career success	0.116	0.033	0.14	−0.03	−0.02	**0.60**	0.01	4.9	1.3
31. Seeking academic success	−0.103	0.16	0.02	0.02	0.20	**0.55**	0.11	5.0	1.3
32. Having a respectable social status	0.106	−0.006	0.05	−0.01	0.04	**0.53**	0.00	4.2	1.5
Factor 7: Communication style									
33. Being able to implicitly express one’s opinions and emotions when in disagreement with others	−0.021	0.005	0.00	−0.01	0.02	−0.02	**0.95**	5.1	1.2
34. Being capable of expressing one’s emotions and opinions appropriately	0.09	0.004	0.03	0.07	0.01	0.05	**0.67**	5.3	1.2
35. Being able to avoid interpersonal conflict with others	0.046	0.18	0.05	−0.02	0.04	0.16	**0.35**	5.1	1.3

*N = 529. Bold values represent factor loadings exceeding 0.35. Using the varimax for orthogonal rotations.*

Factor 1 (11 items) was labeled *Expected Self*. It reflects the values of emotional regulation, daily life functioning, mental strength and resilience, balance between mental and physical wellbeing, and consistency between private and social life. The highest loading items were, “being aware of one’s own negative emotions” and “adjusting to changes at different life and developmental stages.”

Factor 2 (7 items) was labeled *Relating to Others*. It describes the value of connecting and maintaining harmonious relationships with others. All items emphasize what an individual self can contribute to others’ welfare, and how an individual self can relate to others, such as “being capable of loving others” and “feeling gratitude.” The highest loading items were, “bringing positive energy to others” and “helping people in need.”

Factor 3 (3 items) was labeled *Life Principle*. This factor reflects the Confucian value of being modest and prudent. Modesty is one of the core values in Chinese culture, which not only teaches unpretentiousness and avoiding bragging but also promotes humbleness in relating to others. The highest loading items were “being modest” and “being prudent.”

Factor 4 (4 items) was labeled *Family*. This factor demonstrates the value of one’s family, especially having a close relationship with parents. The highest loading items were, “having healthy parents and family members” and “having a harmonious family.”

Factor 5 (4 items) was labeled *Purpose and Meaning*. This factor reflects the value of pursuing purpose and meaning in life. The highest loading items were “having clear goals in life” and “being self-aware.”

Factor 6 (3 items) was labeled *Achievement*. This factor reflects the value of achieving success in career, academic and social status. The highest loading items were “seeking career success” and “seeking academic success.”

Factor 7 (3 items) was labeled *Communication Style*. This factor reflects value of a high-context communication style (i.e., implicit and indirect) and behaviors (i.e., accepting criticism and not deviating from the norm set by the mainstream). The highest loading items were “being able to implicitly express one’s opinions and emotions when in disagreement with others” and “being capable of expressing one’s emotions and opinions appropriately.”

Gender differences were calculated for each item and each factor using Sample B (*n* = 529, 365 females, 164 males), which resulted in significant difference in six items spread across 6 factors (items 3,15, 19, 28, 30, 33). Meanwhile, two genders differ in the overall score of Factor 6 (Achievement), showing that male students placed higher values (*M* = 4.8, SD = 1.0) on achievement than female students (*M* = 4.6, SD = 1.1).

### Confirmative factor analysis

A confirmatory factor analysis, using the maximum likelihood estimation method, was conducted on the 35-item Chinese Mental Health Value Scale for Sample B (*n* = 529). The seven-factor model indicated the following fit indices: *χ*^2^(539) = 1,179.12, CFI = 0.91, TLI = 0.90, SRMR = 0.05, RMSEA = 0.05, 90% confidence interval (0.04, 0.05). Due to the low indices of TLI, modification provided by Mplus output was considered to improve the indices based on theoretical and statistical justifications (Muthén and Muthén, 2007). Under careful examination, four modifications were included in the final CFA model because of correlations between four pairs: items 2 and 1 (factor 1), items 38 and 39 (factor 3), items 33 and 49 (factor 4), and items 64 and 65 (factor 2). As a result, the final 7-factor model with modifications indicated good fit indices, *χ*^2^ (535) = 1,009.08, CFI = 0.93, TLI = 0.92, SRMR = 0.04, RMSEA = 0.05, 90% confidence interval (0.04, 0.05).

### Reliability

Reliability estimates were calculated for Sample A (*n* = 524) and Sample B (*n* = 529) respectively. The internal consistency coefficients for the subscales of the CMHVS and the whole scale were acceptable to very good. For Sample A, internal reliability (Cronbach’s alpha) was 0.96 for the whole CMHVS and 87, 0.88, 0.84, 0.83, 0.85, 0.72, 0.81 for its seven subscales, respectively. For Sample B, the internal reliability was 0.95 for the total scale and 0.87, 0.87, 0.81, 0.80, 0.82, 0.70, 0.77 for its seven subscales, respectively. The seven latent factors were strongly correlated with each other, ranging from 0.51 to 0.85. Correlations between the factors were presented in Table 4.

**Table 4:** Correlations among seven factors of CMHVS.

Factor	1	2	3	4	5	6	7
1. Expected self	—						
2. Relating to others	0.77**	—					
3. Life principles	0.77**	0.85**	—				
4. Family	0.62**	0.85**	0.72**	—			
5. Purpose and meaning	0.65**	0.78**	0.72**	0.68**	—		
6. Achievement	0.61**	0.60**	0.76**	0.51**	0.58**	—	
7. Communication	0.68**	0.74**	0.70**	0.61**	0.66**	0.61**	—
*M*	5.45	5.47	5.22	6.10	5.53	4.67	5.15
SD	0.78	0.90	1.10	0.89	1.00	1.10	0.99

CMHVS, Chinese Mental Health Value Scale.

**Correlation is significant at the 0.001 level.

### Convergent validity

Validity of CMHVS was tested by examining its correlation with AVS, SWLS, COS, and BSI-18. Results were shown in Tables 5, 6. As expected, the total score and seven subscale scores of CMHVS were all positively correlated with AVS and SWLS. Furthermore, the seven subscales of CMHVS were positively and significantly correlated with all four subscales (HI, VI, HC, and VC) of COS, the magnitude of the correlations was consistently larger for Collectivism-related than Individualism-related subscales, and the peak correlation appeared between Horizontal Collectivism and CMHVS. There was no significant correlation between the BSI-18 and CMHVS.

**Table 5:** Intercorrelations among subscales of CMHVS and five referent measures.

		1	2	3	4	5	6	7	8	9	10	11	12	13	14	15	16	17
1	Expected self	—																
2	Relating to others	0.79**	—															
3	Life principles	0.68**	0.74**	—														
4	Family	0.65**	0.80**	0.65**	—													
5	Achievement	0.65**	0.71**	0.72**	0.62**	—												
6	Purpose and meaning	0.69**	0.80**	0.60**	0.67**	0.67**	—											
7	Communication	0.68**	0.73**	0.62**	0.57**	0.68**	0.67**	—										
8	AVS_norm	0.21**	0.23**	0.18**	0.31**	0.19**	0.18**	0.14**	—									
9	AVS_humility	0.35**	0.45**	0.53**	0.37**	0.31**	0.31**	0.33**	0.28**	—								
10	SWLS	0.18**	0.22**	0.15**	0.11**	0.15**	0.12**	0.15**	−0.01	0.18**	—							
11	COS_HI	0.33**	0.33**	0.31**	0.22**	0.40**	0.35**	0.33**	−0.17**	0.31**	0.17**	—						
12	COS_VI	0.32**	0.31**	0.34**	0.26**	0.57**	0.29**	0.31**	0.08	0.30**	0.07	0.84**	—					
13	COS_HC	0.49**	0.55**	0.44**	0.44**	0.47**	0.41**	0.43**	0.21**	0.52**	0.33**	0.45**	0.44**	—				
14	COS_VC	0.29**	0.38**	0.36**	0.47**	0.42**	0.27**	0.29**	0.27**	0.49**	0.19**	0.43**	0.49**	0.71**	—			
15	BSI_Anxiety	−0.07	−0.02	−0.02	−0.03	0.04	0.01	−0.04	−0.13*	−0.12*	−0.34**	0.01	0.09	−0.19**	−0.11**	—		
16	BSI_Soma	−0.12**	−0.01	−0.02	−0.07	0.04	−0.02	−0.03	−0.14*	−0.19**	−0.31**	0.01	−0.01	−0.16**	−0.13**	0.82**	—	
17	BSI_Depre	−0.08*	−0.03	−0.04	−0.05	0.03	0.00	−0.05	−0.12*	−0.16**	−0.43**	0.04	0.13**	−0.21**	−0.11*	0.95**	0.79**	—

CMHVS, Chinese Mental Health Value Scale; 1–7, seven factors of CMHVS; AVS, Asian Value Scale; AVS_norm, AVS_Conformity to Norm; SWLS, Satisfaction with Life Scale; COS, Cultural Orientation Scale; HI, Hierarchical Individualism; VI, Vertical Individualism; HC, Hierarchical Collectivism; VC, Vertical Collectivism; BSI, Brief Symptom Inventory. *p < 0.05, **p < 0.01.

**Table 6:** Correlations among total scores of CMHVS and referent measures.

	CMHVS	AVS	SWLS	COS_Indi	COS_Coll	BSI
CMHVS	—					
AVS	0.29**	—				
SWLS	0.14**	−0.03	—			
COS_Indi	0.32**	0.04	0.08*	—		
COS_Coll	0.45**	0.28**	0.25**	0.34**	—	
BSI	−0.05	−0.14*	−0.31**	0.07*	−0.15**	—

CMHVS, Chinese Mental Health Value Scale; AVS, Asian Value Scale; SWLS, Satisfaction with Life Scale; COS, Cultural Orientation Scale; COS_Indi, COS_Individualism; COS_Coll, COS_ Collectivism; BSI, Brief Symptom Inventory.

*p < 0.05, **p < 0.01.

## Discussion

As expected, the study results offered knowledge and insight regarding Chinese college students’ mental health values and produced an evaluation scale with sound psychometric qualities. The CMHVS shows high reliability with Cronbach’s alpha of 0.96, and convergent validity with its high positive correlations with AVS, SWLS, and COS. From a cultural perspective, this scale tightens the connection between mental health and cultural values. Further, it offers support to practice that aims at promoting culturally supported and functional mental health for Chinese college students.

### Mental health as a culturally informed phenomenon

Consistent with the principle of cultural psychology, “One mind, many mentalities” (Shweder et al., 1998), CMHVS sheds light on both the universal mind of human beings and the culturally specific mentality regarding mental health for Chinese colleges students. CMHVS shares some commonalities with the available mental health value scales in the U.S. (Jensen and Bergin, 1988; Kelly, 1995; Tyler et al., 1983). The most obvious is that all scales contain largely positive concepts and desirable behaviors, implying that mental health is a positive construct. For instance, shared subscales include interpersonal relationship, emotional control/regulation, and personal success/achievement. Although there are differences in the specific content or scope/depth of each area across measures, these subscales nonetheless reflect the common and basic dimensions of human mental experience.

Differences between CMHVS and western scales are notable as well. First, CMHVS appeared to reflect collectivistic/relational values more and individualistic values less than the existing western scales. Expectedly Chinese students failed to show high value of the western cultural ideals such as independence, autonomy, self-reliance, and explicit and direct communication, which are the foundations of western psychology. Because Chinese are more likely to define self-in-relations, and a collectivistic/relational orientation is more discernable in CMHVS than in other scales. The psychological anthropologist Alan Page Fiske famously categorized human interactions into four types, communal sharing, authority ranking, equality matching, and market pricing (Fiske, 1991). The values in CMHVS seem to reflect more communal sharing (e.g., “bringing positive energy to others”) and authority ranking (e.g., “showing filial piety to parents”) and less equality matching and market pricing than existing western scales. The Confucian social relationism is shown throughout the scale especially in the subscales of *Expected Self*, relation to others, and communication. The others-focus mentality is clear and salient in how one expects the self to take other’s perspective and stay in harmony with and being a giver/helper to others.

Secondly, the highly valued emotional expression and taking active actions for change in western cultures are weak in CMHVS. Instead, a status of *being* is salient in “having a peaceful mind,” “being modest,” “feeling gratitude,” “feeling satisfied,” or “being modest.” The Chinese folk wisdom values one’s capacity to control negative emotions and accept life adversities as a way of dealing with stress. Additionally, values based in dialecticism emphasize the balance and harmonious states in physical, emotional, interpersonal and spiritual life, such as “being neither arrogant about winning nor discouraged after losing,” etc. Such passive ways of being and relating to the world may not fit dominant western values but certainly contribute to peaceful mind maintenance and conflict avoidance for Chinese.

Indeed, CMHVS portrays that it is the culture that determines what traits, emotions, and behaviors would be experienced as healthy or unhealthy. The *guanxi* (relationship) orientation, *he* (harmony) mentality, and *jia* (family) centrality are distinctively reflected in multiple subscales. Rather than focusing on independence and autonomy as valued by western cultures, the *Expected Self* includes being able to take others’ perspective, building harmonious relationship with others and managing all relationships in life to maintain harmony. Being altruistic and grateful to and loving others as well as bringing positive energy to others are valued as ways of relating to others. These values reflect the self-in-relation framework as a key expression of Chinese relationism (Hwang, 2012).

### Cultural values, mental health values, and psychological wellbeing

The study result showed that participants who held higher Asian values endorsed CMHVS more, meaning mental health values are consistent with general cultural values. Moreover, the seven subscales of CMHVS were positively correlated with all four subscales of Collectivism–Individualism Scale, showing that individualism and collectivism are not dichotomous but co-existent for contemporary Chinese college students, although participants reported higher collectivism than individualism. Notably, the peak correlations exist between Horizontal Collectivism (emphasis on sociability and interdependence) and almost all subscales of CMHVS, which shows that Chinese college students highly value relationships. The high correlations between both Hierarchical and Vertical individualism scales and *Achievement* (Factor 5) of CMHVS, however, seemed to show that participants were aware that they need to work hard and be competitive to succeed or excel in today’s environment where academic stress is extremely high.

As expected, CMHVS was positively correlated with life satisfaction, which means that Chinese college students’ mental health values are functional to them in increasing life satisfaction. This relationship becomes even more significant when considering the lack of correlation between CMHVS and psychological symptoms measured by BSI-18 except for the negative correlation between Somatization and Depression and *Expected Self* (Factor 1). It is worth noting that many items of *Expected Self* describe personal strengths and resilience, such as being aware of one’s negative emotions, controlling one’s negative emotions, and accepting adversities in life. Likely, such personal strengths may prevent depression and somatization symptoms. Compared to Western scales like the Mental Health Continuum–Short Form (MHC-SF; Keyes, 2005), which emphasizes autonomy and self-acceptance, the CMHVS highlights relational and familial dimensions of wellbeing. While both scales capture psychological flourishing, CMHVS uniquely integrates Confucian ideals of self-cultivation and collective harmony, providing a more culturally relevant measure for Chinese students.

### Characteristics of college students’ mental health values

China is believed to have experienced large and transformative cultural changes in recent decades, including rising importance of materialism, individualism, freedom, democracy (Xu and Hamamura, 2014). Interestingly, however, the values captured by CMHVS generally do not show clear support to this perceived trend but back the enduring theme of family and relationship. Students’ adherence to traditional cultural values in viewing mental health is apparent. The values such as relationship, harmony, filial piety, and context-driven communication are underlying their mental health values in *Expected Self*, *Relating to others*, *Family*, and *Communication* subscales.

However, indirect support for modernization of values can be seen through the positive correlations between the individualism subscales of COS and seven factors of CMHVS. Perhaps in college students’ mind independence, autonomy, self-reliance, and other individualistic concepts are important for them if they want to be successful. The item “taking responsibility for the consequences of one’s choices” fits the traditional value of being a responsible and accountable person on one hand and is consistent with the individualistic notion of being self-reliant on the other. *Achievement* is an example of Chinese college students, especially male students, operating in the mist of both the old and new value systems.

### Implications for practice and research

The CMHVS established a solid connection between Chinese college students’ mental health values and their cultural contexts. Using it as an assessment tool or as a reference, researchers and clinicians may be reminded that mental health promotion for Chinese students needs to be examined with full consideration of their values.

For clinicians trained with western theories in China or elsewhere, this study can inform them that conceptualization of clients should not be dictated by the western theories. Some valued behaviors may not appear healthy in a western cultural context or by western theoretical perceptions, but needed to be validated and confirmed because they are correlated with wellbeing for Chinese students. When Chinese students present themselves as weak in ego strength or low in self-esteem, clinicians needs to be reminded that the self-in-relation or others-focused relationship is the essence of being for those students. The importance of emotional balance in academic settings has been emphasized in previous research (Diotaiuti et al., 2021), which found that procrastination and emotional self-regulation significantly impact students’ performance. Our findings align with this perspective, highlighting the role of mental health values in promoting psychological wellbeing and academic success. CMHVS may be helpful to gaining an understanding their mental health values to avoid premature judgment. Furthermore, practitioners need to be aware of their own values, the values embedded in their theoretical orientations, and their student clients’ values to avoid imposing irrelevant values or overriding values of their clients. CMHVS may provide a tool for clinicians to take into consideration of “balancing physical and mental wellbeing” and “balancing one’s private life and social life” for their Chinese clients.

CMHVS also provides a research tool for understanding Chinese college students’ mental health needs and exploring effective educational/preventive/interventional methods for this population. Specific to counseling, future research may exam the relationship between mental health values and Chinese client in-session behaviors, including openness to various therapeutic interventions. Additionally, it may be meaningful to explore whether and to what extent Chinese students would use those values to monitor their behaviors and guide their daily life. Due to the fast-increasing number of Chinese international students in the United States or other countries, researchers may also use CMHVS to examine its cross-sample validity and its association to these students’ acculturation and cultural identity.

### Limitations

First, although the sample size of the study was large enough, there was a methodological limitation that the data were randomly split into two samples for EFA and CFA. Thus, the two samples may reflect similar sampling bias, measurement bias, or experimenter effects. Second, the current study did not directly measure western mental health values among Chinese students and compare them with Chinese ones, thus future research can incorporate mental health value scales developed based on western culture. Third, although most reverse items were proportionately represented by other non-reverse items, it was a loss of information to remove them in EFA due to the responding patterns. It is likely that when measuring values, reverse items may cause confusion about how to give precise answers. Fourth, the current research only used CFA in scale validation, while other psychometric methods can also be included for future research such as classical test theory, item response theory, and Rasch model theory (see Xu et al., 2020, for an example). Fifth, given the emphasis on relational and moral values, some participants may have responded in ways that reflect social expectations rather than personal beliefs. Future studies could incorporate social desirability scales to control for this bias. Finally, while this study establishes the CMHVS as a reliable research tool, its clinical utility remains to be tested. Future studies should examine its application in therapy settings to assess its impact on treatment outcomes.

## Data Availability

The raw data supporting the conclusions of this article will be made available by the authors without undue reservation.
